# Barriers and Facilitators of Platform Trials

**DOI:** 10.1001/jamanetworkopen.2026.3758

**Published:** 2026-04-02

**Authors:** Stuart McLennan, Alexandra Griessbach, Sharon Love, Christof Manuel Schönenberger, Alain Amstutz, Lucille Sebastian, Dominique Costagliola, Inge Christoffer Olsen, Corina Silvia Rueegg, Matthew R. Sydes, Matthias Briel, Benjamin Speich

**Affiliations:** 1 CLEAR Methods Center, Division of Clinical Epidemiology, Department of Clinical Research, University Hospital Basel, University of Basel, Basel, Switzerland; 2 Institute of History and Ethics in Medicine, Department of Preclinical Medicine, TUM School of Medicine and Health, Technical University of Munich, Munich, Germany; 3 MRC Clinical Trials Unit, Institute of Clinical Trials and Methodology, University College London, London, United Kingdom; 4 Population Health Sciences, Bristol Medical School, University of Bristol, United Kingdom; 5 Oslo Centre for Biostatistics and Epidemiology, Oslo University Hospital, Oslo, Norway; 6 NHMRC Clinical Trials Centre, University of Sydney, Sydney, NSW, Australia; 7 Sorbonne Université, INSERM, Institut Pierre Louis d’Épidémiologie et de Santé Publique, Paris, France; 8 Deptartment of Research Support for Clinical Trials, Oslo University Hospital, Oslo, Norway; 9 Epidemiology, Biostatistics & Prevention Institute, University of Zurich, Zurich, Switzerland; 10 Department of Health Research Methods, Evidence, and Impact, McMaster University, Hamilton, Ontario, Canada

## Abstract

**Importance:**

Platform trials have emerged as a novel approach to clinical trials and are often described as a more resource- and cost-efficient design. Despite their growing relevance, the experiences and challenges of teams conducting platform trials remain underexplored.

**Objectives:**

To examine the experiences and views of platform trial teams regarding the barriers and facilitators raised when conducting platform trials.

**Design, Setting, and Participants:**

This cross-sectional survey study was carried out from June to August 2023 and was analyzed with a mixed-methods approach. The survey was sent to 127 principal investigators of platform trials and 11 other stakeholders who are experienced in the methodology and conduct of platform trials. The survey could be forwarded to other relevant team members of a platform trial.

**Main Outcomes and Measures:**

Survey data on barriers and facilitators to conducting platform trials were analyzed using descriptive statistics and standard qualitative content analysis.

**Results:**

A total of 40 completed surveys were returned. Respondents’ roles in platform trials included trial management (22 respondents [55%]), principal investigator (12 respondents [30%]), statistics and trial methodology (11 respondents [28%]), and data management (11 respondents [28%]). Respondents reported efficacy gains from using a shared coordinating infrastructure for multiple intervention arms as the key advantage of platform trials, although these require more planning time and greater team expertise than traditional randomized clinical trials. There was no consensus on whether the perceived costs and resources needed for adding a comparison within a platform trial were lower or higher compared with setting up a traditional randomized clinical trial. A repeatedly raised challenge was that current regulatory and organizational processes are set up for intervention-specific clinical trials and are incompatible with adaptive and open-ended platform trials. Targeted supports such as guidelines, templates, funding mechanisms, and infrastructure setup for platform trials were identified as facilitators for their conduct.

**Conclusions and Relevance:**

In this survey study of platform trial experts, it was found that the clinical research landscape is currently not set up for adaptive platform trials, which leads to substantial challenges conducting such trials. These findings suggest that stakeholders need to take concerted action to enable the integration of platform trials.

## Key Points

**Question:**

What barriers and facilitators do teams encounter when conducting platform trials?

**Findings:**

In this survey study of 40 investigators experienced in conducting platform trials, respondents valued platform trials’ efficiency from shared infrastructure but noted greater planning time and need for expertise and training compared with traditional randomized trials. Perceived costs and resource requirements were uncertain, and regulatory and organizational processes were often incompatible with platform trials’ adaptive and open-ended designs.

**Meaning:**

These findings suggest that successful integration of platform trials into clinical research requires better-adapted processes to appropriately and efficiently implement them when needed.

## Teaser

This survey study examines the experiences and views of platform trial teams regarding the barriers and facilitators raised when conducting platform trials.

## Introduction

Randomized clinical trials (RCTs) play an essential role in the evaluation of health care interventions.^1,2^ Typically, an RCT is set up to answer one specific scientific question about one single intervention. However, RCTs are usually time consuming and resource intensive.^3,4^ Setting up a new RCT each time there is an intervention to assess can be slow, inefficient, and costly.^5,6^ Platform trials have emerged as a novel family of approaches to traditional RCTs, with no uniformly agreed-upon description. One study described them as:

Platform trials are RCTs that allow for multiple interventions to be simultaneously evaluated and new interventions to be added after the trial is initiated. The platform provides a single infrastructure wherein interventions can be added and discontinued at different times after their clinical questions are answered through comparisons against a control group, sometimes shared across interventions.^7^

A systematic review found that there was a doubling in the initiation of platform trials at the beginning of the COVID-19 pandemic, albeit from a low baseline.^8,9^ Although the number of newly initiated platform trials has since declined, they will continue to play an important role in clinical research. However, platform trials are complex and understanding of the challenges involved in conducting them is limited. Furthermore, platform trials are often described as a more resource- and cost-efficient approach, but evidence to substantiate these claims has been largely limited to simulation studies.^10,11^

High operational complexity in platform trials has been noted, with protocol structure, database setup, and the difficulties in adding and removing comparisons without affecting ongoing comparisons, being particularly emphasized.^12,13,14,15^ In terms of practical experience, the FOCUS4 platform trial team has described various challenges that arose and lessons learned over a 10-year journey.^16^ These challenges included predicting the actual resource capacity required for platform trials, finding suitable and adequate funding, underestimating the required staffing and workload, and issues related to the engagement of pharmaceutical companies.^16^ In a series of articles, the Systemic Therapy in Advancing or Metastatic Prostate Cancer: Evaluation of Drug Efficacy (STAMPEDE) trial team also shared their insights on the complexities of platform trial statistics, data management, the operational burden for the trial team, and the challenges encountered when adding new treatment arms.^12,13,14,17^ Nevertheless, besides these few examples, there is a lack of research on the experiences of platform trial teams on a global scale. We therefore invited representatives from all existing platform trials to assess their experiences and views regarding the barriers and facilitators involved in conducting platform trials. This will potentially help identify shortcomings in current clinical trial infrastructure and processes and assist in informing standard practice guidelines.^12,13,14,15^

## Methods

This survey study follows the Consensus-Based Checklist for Reporting of Survey Studies (CROSS) reporting guideline.^18^ It is also guided by the Standards for Reporting Qualitative Research (SRQR) reporting guideline in relation to the qualitative analysis conducted.^19^ The study received a waiver from review from the Ethics Committee of Northwestern and Central Switzerland because no personal health-related data were collected in accordance with the Common Rule.

### Research Team and Reflexivity

In the spirit of reflexivity, we would like to situate ourselves in relation to platform trials. The research team consists of academic researchers (5 women and 7 men) who conduct meta-research on clinical research methods (A.G., C.M.S., A.A., M.B., and B.S.), including a number of people who have been actively involved in the development and conduct of platform trials (S.L., L.S., D.C., B.S., M.B., I.C.O., C.S.R., and M.R.S.). It also includes a senior interdisciplinary bioethicist (S.M.) who has extensive experience with qualitative research. The research team had already had contact with several of the platform trial teams invited to participate in the survey. Otherwise, no relationship was established between the research team and the other participants prior to the study. There was no hierarchical relationship between the research team and the survey respondents.

### Survey Contents

The survey was developed by the research team through several rounds of discussion and consideration (eAppendix in Supplement 1). Survey questions first explored respondents’ roles and platform trial expertise. Respondents were then asked free-text questions about their experiences with challenges at different stages of platform trials (regulatory processes, funding acquisition, planning and setup, patient consent and information, and patient and public involvement, clinical trial registries, conduct, data analysis, and results dissemination) and to specify potential solutions. Finally, respondents were asked about the most important advantages of platform trials, particularly compared with traditional RCTs, including time to plan, time to add new comparisons, study team expertise and training requirements, costs and resources needed, and time until the first participant is randomized. Respondents’ agreement was assessed on a 5-point Likert Scale.

### Survey Implementation

The cross-sectional survey was conducted using REDCap^20,21^ and responses were anonymous. The survey was pilot tested with 6 platform trial experts from 1 platform trial team before mass distribution. After pilot testing, some phrasing of the questions was revised for clarity. The survey was then sent to the principal investigators of all 127 platform trials previously identified in a systematic search.^8^ We also contacted a purposeful sample of 11 other stakeholders from the research team’s network who are experienced in the methodology and conduct of platform trials. Survey recipients were asked to fill out the survey and to forward the invitation link to their platform trial team members or other experts on platform trials. Filling out and submitting the survey was considered informed consent. The survey was sent to potential respondents via email, and 4 reminders were sent out after 7, 14, 30, and 60 days. The survey was carried out from June to August 2023.

### Data Analysis

The survey data was analyzed descriptively using R version 4.1.3 (R Project for Statistical Computing). Quantitative data are reported as absolute numbers and percentages. For multiple-choice questions using the Likert Scale, the denominator was adjusted to account for missing data. The original free-text responses were analyzed with conventional qualitative content analysis.^22^ Initial themes were labeled in team meetings (S.M.L., A.G., C.M.S., M.B., and B.S.) using a process of open coding, focusing on themes common across respondents as well as those unique to individuals that may offer insight into differences in perspectives. Conversations among the investigators continued until coding differences were resolved and consensus was achieved. Findings are presented as higher- and lower-level categories in a coding frame, with example quotations provided.

## Results

### Respondent and Platform Trial Characteristics

A total of 40 experts agreed to participate in the study and answered the survey between June and August 2023. The overall response rate cannot be calculated; because the survey could be forwarded to additional team members and colleagues, there is uncertainty how many people received the invite. However, 138 potential respondents were formally invited (ie, 127 principal investigators plus 11 additional experts), so the response rate was no higher than 30%. Respondents were involved in a total of 36 different platform trials (32 from the sample of 127 previously identified platform trials in 2022 and an additional 4 newly identified platform trials). The platform trials that were represented by respondents were all non–industry funded, and the majority were COVID-19 platform trials (16 of 32 trials [50%]) or oncology platform trials (8 of 32 trials [25%]) (eTable 1 in Supplement 1). The 40 respondents had various, and often concurrent, roles in relation to platform trials, including trial management (22 respondents [55%]), principal investigator or supervisor (12 respondents [30%]), statistics and trial methodology (11 respondents [28%]), data management and programming (11 respondents [28%]), monitoring (9 respondents [23%]), regulatory affairs (9 respondents [23%]), patient and public involvement (3 respondents [8%]), and site investigator (1 respondent [3%]). The majority of respondents (24 respondents [63%]) reported that they had central organizational expertise, 13 respondents (34%) reported both central organizational expertise and site-specific expertise, and 1 respondent (3%) reported only site-specific expertise.

### General Advantages and Disadvantages of Platform Trials

The efficiency that came by having a shared coordinating infrastructure with a single comparator arm for multiple intervention arms compared with setting up multiple individual trials, was repeatedly identified by respondents as the key advantage of platform trials (Table 1). This was also seen to have secondary advantages, including helping to reduce research and resource waste, having long-term cost and time benefits, faster recruitment, and having increased speed and ease of opening new intervention arms. Another important advantage of platform trials identified was the improved retention of knowledge in the study team.

**Table 1.** Advantages of Platform Trials

Code	Example quotations
Efficiency of a shared infrastructure	“The efficiency. I’m referring both to the efficiency of being able to share controls and/or information across subprotocols and the efficiency of resource use.” (P21)
	“The efficiencies. From a numbers perspective, it just kind of makes so much sense. For example, fewer patients are allocated to the control group, the master protocol stays the same. There’s just so much efficiency gained. Once you have all of those component pieces in place, you’re not breaking it down at the end and building it from scratch. You’ve already built that infrastructure one time. So, the start-up costs are probably quite similar BUT I’m not wasting money at the back end by deconstructing it and building it up again like in regular RCTs. I just keep using the same infrastructure, and it’s faster than a regular RCT.” (P40)
Reduced waste	“Fast failure of treatments limits research waste.” (P37)
Cost and time benefits	“Long term cost benefits and time benefits.” (P15)
	“Can answer questions faster and with better returns on investment and once set up and all the energy required for this then then can continue to operate in perpetuity.” (P39)
Faster recruitment	“Faster recruitment.” (P3)
Speed and ease of opening arms	“Once the platform is up and running, relatively straight forward to keep enrolling patients and add/drop interventions.” (P34)
Knowledge retention of study team	“The knowledge gained by study team was passed from one arm to the next and reduced the time needed to get into the trial.” (P2)
	“Platforms can be enduring, limiting loss of knowledge through loss of trials professionals at end of study.” (P37)

Abbreviations: P, participant; RCT, randomized clinical trial.

Although stakeholders saw these advantages of platform trials over traditional RCTs, 89% of stakeholders (33 of 37 respondents; 3 respondents with no response) reported that planning platform trials takes much more time or more time than planning traditional RCTs, and 82% (31 of 38 respondents; 2 respondents with no response) stated that study team expertise and training required for platform trials is much higher or higher than traditional RCTs (Figure and eTable 2 in Supplement 1). The time for planning the addition of a new comparison to a platform trial compared with the time needed to plan a standalone trial was judged as less or much less by 54% of respondents (20 of 37 respondents; 3 respondents with no response), however, 22% (8 of 37 respondents) stated that it seemed to take more time. Respondents showed no clear consensus if (1) the perceived time needed until the first patient is recruited and (2) the amount of costs and resources needed were higher for a platform trial or traditional RCT (Figure).

**Figure.**
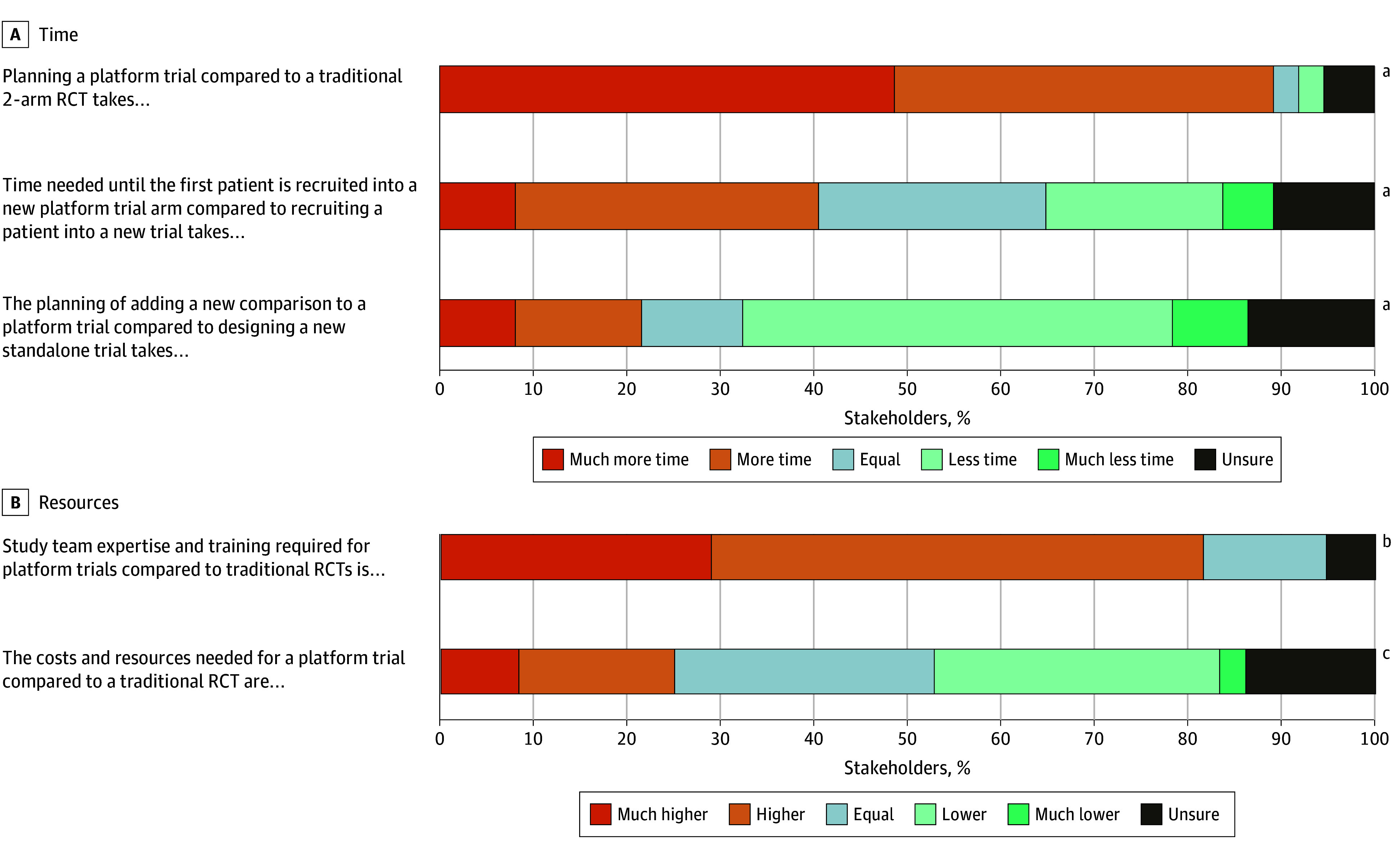
Stacked Bar Graphs Comparing Time and Resource Use of Platform Trials vs Traditional Randomized Trials ^a^Missing 3 respondents. ^b^Missing 2 respondents. ^c^Missing 4 respondents. RCT indicates randomized clinical trial.

### Specific Platform Trial Challengesand Recommendations

Respondents identified a range of specific challenges at different stages of platform trials. Respondents also provided recommendations to address these challenges (Table 2 and eTable 3 and eTable 4 in Supplement 1).

**Table 2.** Challenges of Platform Trials

Codes and subcodes	Example quotations
Planning and setup (24/40 [60%])	
Challenges	
Complex legal arrangements	
Multiple contract negotiations need a lot of time	“Multiple contracts for the overall trial and on addition of arms.” (P12)
	“Multiple pharma companies were involved with the study; contract negotiation took longer than for a standard trial.” (P20)
Difficulty obtaining trial insurance on open-ended trial	“Obtaining the proper clinical trials insurance can be another challenge, logistic-wise. You cannot write an application for the insurances with no end date. These applications aren’t built for this trial design because this trial “never ends” or does not end until we find a really effective treatment. We’re forced to pick an end date in the register too.” (P40)
Difficulties writing standard operating procedures for dynamic trials	“How do you write a standard operating procedure form of a protocol that you don’t know what it’s going to look like in years from now? That can be very challenging.” (P40)
Difficult to set up databases that are flexible enough for platform trials	“Database set up as been incredibly difficult—ie, building a database that can manage the complex framework of a platform trial while at the same time trying to make it streamlined for site staff collecting and entering the data.” (P8)
	“The database and, in particular, the randomization needed to be flexible enough to allow the addition of subprotocols.” (P21)
Recommendations	
Standardizing contracts	“Template contracts specifically for platform trials.” (P12)
	“Ideally would be great if pharma companies would agree to use standard template for academic trial—templates for these do exist in UK, however[…]preferences for many pharma companies is to use their own template.” (P20)
Centralized information and resources to help set up platform trials	“Having centralised information and resources to help platform trials set up and manage their trials—rather than having to research and find the relevant information themselves—need to prevent various platform trials from reinventing the wheel each time.” (P8)
Regulatory processes (21/40 [53%])	
Challenges	
Increased regulatory complexity	
Regulatory complexity	“Ethics renewals and contract renewals are difficult and time consuming. Due to multi-arm nature of platform trials, conduct is more challenging than conducting traditional trials (eg, 2-arm trials).” (P15)
	“This is an ongoing challenge as you learn to operate in a highly complex and dynamic environment, particularly when it is international and each jurisdiction has specific rules that require adaptation.” (P39)
Lack of expertise	“Authorities did not have the expertise for this type of trial.” (P2)
Incompatible regulatory processes	“New [clinical trial registries] are not flexible enough for platform trials.” (P36)
	“Addition of treatment arms was seen [as] a new trial by some countries regulatory authorities.” (P14)
Recommendations	
Updated regulations	
Clear guidance	“Clear guidelines by authorities for dealing with new adaptive designs.” (P2)
Better coordination between ethics committees	“Modification of the CTIS specially for the amendment submission but also for a better coordination between the national ethics committee.” (P16)
Specialized ethics committees	“Specialist ethics committees for adaptive designs…” (P13)
Fast-track systems	“Fast track systems for platform trials would be beneficial to prevent delays in adding/removing arms.” (P29)
Conduct (20/40 [50%])	
Challenges	
Insufficient resources for the open-ended format	
Not enough funds and time to add new arms	“Insufficient resources and time to continue with a trial format to add new arms to the trial.” (P3)
Not enough staff	“We have limited resourcing in terms of project staff.” (P8)
	“The sites look at a platform trial as one trial. So they staff it as one trial. But this is a lot more work because this is a lot more drugs, and because you’re always putting patients in, sites tend to have more patients than they would on a standard protocol. These are very ill patients that require a lot of care by the physicians and all the support staff. It’s a lot of work, and it’s changing the mindset of how do I even make the calculations of how much staff hours to use to support the study.” (P40)
Operational complexity	
Adding new arms	“Scientific ideas pushed but operations not considered and then have to be adapted to adjust to science, eg, including blinding and adding additional arms and how it is managed.” (P17)
Managing complex randomization	“Managing the complexity of the different randomised pathways, multiple randomisation events.” (P9)
Managing drug supply	“Drug supply—must have individualized approaches to each company partners, while also maintaining standardization across all processes (eg, standardized contracts, etc).” (P7)
Complex data management	“Multiple adaptations for database. Difficult for sites to keep on top of changes and updates to documentation.” (P19)
Lack of expertise	“It can be a challenge to communicate changes to the platform trial especially when sites haven’t worked on one before.” (P29)
	“Platform trials aren’t easy to run, and it’s hard to explain them to others because they’re not familiar with this design. The people who do regular clinical trials know the standards and expectations that you can have. But then people are very confused about what we’re doing because we’re doing it completely differently.” (P40)
Overburdened staff	
Staff overwhelmed	“Study nurses were exhausted and glad when the trial finally stopped. Adding arms constantly does not give reprieve.” (P2)
	“With a trial like this, there’s never that downtime. Just as you’re getting to the point where you might be closing out an arm, you’re bringing in more arms.” (P40)
Staff turnover	“Turnover of human resources.” (P23)
Recommendations	
Ensuring sufficient resources	
Accurate budgeting of trial	“Cost the trial appropriately and allow sufficient funds for additional personnel and support…” (P17)
Specialized teams	“Having dedicated teams who have expertise on platform trials eg, database teams, stats teams, project management teams.” (P8)
Improving support and training of study team	“Support of study team on site. Clear plans and input from study nurses how to improve recruitment of patients and how best to improve their work conditions.” (P2)
	“Education and training for site research professionals.” (P13)
Funding acquisition (16/40 [40%])	
Challenges	
Incompatible funding processes	
Incompatibility	“Almost all of the competitive grants are based on a project-specific basis. Platform trials require long-term funding to sustain over long-term, and funding is not always guaranteed.” (P15)
	“Funder wanted to fund in same way they do a standard clinical trial—they were not set up to support adding/removing arms with the contractual and funding framework they use. They wanted to fund arms that started at the same time (multi-arm study) but not the multi-stage part as their processes did not work for that.” (P28)
Need for multiple grants	“With multiple grants it is difficult to manage.” (P19)
Aligning platform trial with funders timelines	“We had difficulty getting the timing of the launch aligned with funders timelines.” (P21)
Estimating budgets for future arms	“Difficulty to use fundings from different parts, to anticipate the right budget for a new arm.” (P16)
Unequal benefits	“In [country], each grant award is made specific to an academic institution with usually a single PI specified. The specified institution receives indirect cost that supports overhead, and as a result, that PI will receive the credit that benefits their academic career the most over other PIs or co-PIs that may have had important role in the conception, funding acquisition, design, and conduct of the platform trial.” (P15)
Recommendations	
Specific, flexible, ongoing structural funding	“To have a structural sustainable funding to maintain the platform and, if necessary, begin a new arm.” (P16)
Enable platform trials to be multi-institutional	“Ways to improve collaboration between different investigators are needed. Enabling a platform trial to be multi-institutions, instead of a single institution always receiving all of the grant will be important to align the incentives of the academics involved.” (P15)
Clinical trial registries (15/40 [38%])	
Challenges	
Not set up for platform trials	
Not possible to enter complex information	“It was not possible to adequately show the structure of the trial in the registry. Registry is not built for platform trials. The start number of arms, the arms added and stopped, the dates and the number of patients for each arm including number of patients actually recruited, should also be shown on the registry.” (P2)
Requires information to be invented	“We’re forced to pick an end date in the register.” (P40)
Recommendations	
More flexibility	“Option to select platform trial and the flexibility required as arms are added.” (P12)
Patient information and consent (10/40 [25%])	
Challenges	
More complex patient information sheets and consent forms required	“It is much longer than a usual consent.” (P26)
	“People are not used to this approach so more explanation is required.” (P34)
	“Traditional patient information sheets and consent forms do not easily adapt to changes in domains/treatments. Multiple document amendments required…” (P37)
Multiple consents confuse and annoy patients	“Patients needed to reconsent for each subprotocol added. Sometimes they were confused and annoyed, why they needed to sign another informed consent.” (P2)
Recommendations	
Guidelines regarding consent forms	“Having guidelines on how best to set up platform trial consent forms, having generic templates.” (P8)
Patient and public involvement	“Public engagement.” (P32)
Data analysis (9/40 [23%])	
Challenges	
Increased time and complexity	“Much more demanding than traditional trials.” (P15)
	“Volume of data that needs to be cleaned.” (P19)
	“Time for data harvest, data cleaning, data monitoring to perform statistical analysis.” (P23)
Recommendations	
Adequate funding	“Require adequate funding to be allocated to statisticians and statistical programmers to support data analysis.” (P15)
Guidance of handling shared controls	“Guidance on data collection/fields for shared controls, eg, what was available at that site for randomization at the time of enrolment, what they were eligible for randomization at the time of enrolment. Mechanisms for separating that out neatly.” (P13)
Results dissemination (8/40 [20%])	
Challenges	
Disseminating results from one arm while arms are ongoing	“Disseminating the findings from one arm whilst still managing the other arms can be a challenge.” (P29)
Journal editors and reviewers not familiar with platform trials	“Journals and reviewers were less certain about manuscripts reporting platform designs with one comparator arm, in which contemporaneous controls were used for analyses. It was clear several reviewers/editors were not familiar with these designs and were concerned about adaptive platform trials as a concept and had strong preferences for more standard trial designs.” (P28)
Recommendations	
Clearly distinguishing between arms and research questions	“Clear differentiation between arms and research questions.” (P2)
Specific publication guidelines	“Having specific publication guidelines for platform trial protocols as they differ from standard clinical trials.” (P8)

Abbreviations: CTIS, clinical trials information system; P, participant; PI, principal investigator.

#### Planning and Setup 

Of the 40 respondents, 24 (60%) answered that they faced specific challenges at this stage. The planning and setup of platform trials was reported to be challenging due to complex legal arrangements, including negotiations for multiple contracts taking a long time and difficulties obtaining insurance for trials without a clear end date. It was also reported to be challenging to write standard operating procedures and develop databases for platform trials due to their complex and dynamic nature. Respondents felt that the availability of standardized template contracts between sponsors and sites for platform trials would be helpful. Centralized information and resources to help set up platform trials was also advocated for to avoid the need for each platform trial to reinvent the wheel.

#### Regulatory Processes

 Of the 40 respondents, 21 (53%) reported challenges at this stage. Regulatory processes were reported to be typically more complex and time-consuming for platform trials. Key reasons for this were ethics committees’ lack of expertise in handling platform trials, particularly around their adaptive designs, with new comparisons being included and others concluded. It was also noted that current regulatory processes were usually set up for intervention-specific clinical trials and incompatible with platform trials that often involve a master protocol without specified interventions and potentially multiple drug sponsors, and in some cases, the addition of treatment arms was considered a new clinical trial by some regulators. Respondents pointed to the need for updated regulations and clear guidance to deal with new adaptive designs like platform trials. It was suggested that there was a need for better coordination between national ethics committees and that specialized ethics committees and fast-track systems for platform trials would be beneficial to prevent delays and make the process run more smoothly with the opening of additional comparisons and the closure of comparisons.

#### Conduct

Of the 40 respondents, 20 (50%) reported challenges at this stage. Respondents reported that there are often not sufficient resources for platform trials, including insufficient funds (eg, for the platform trial infrastructure as well as for adding new comparisons), time to add new arms, and inexperienced staff at sites. The operational complexity of platform trials was also reported to challenge staff who are more familiar with standard trial designs. This can lead to difficulties around adding new comparisons through the inclusion of new arms, managing complex randomization, managing multiple lines of drug supply, complex data management, and challenges explaining platform trials to potential participants. These challenges and the open-ended nature of platform trials were reported to overburden study staff, with staff becoming overwhelmed, which can lead to higher staff turnover. Respondents highlighted the need for excellent project management and accurate budgets for trials, as well as having specialized and dedicated teams for platform trials who received appropriate support and training. Ensuring sufficient resources for conducting a platform trial was seen as essential.

#### Funding Acquisition

Of the 40 respondents, 16 (40%) reported challenges at this stage. Current funding processes were noted to have limited compatibility with platform trials, with funding usually being project-specific and not set up to support long-term ongoing trials and the adding and removing of arms at different times. The current funding frameworks result in difficulties obtaining multiple grants from multiple funders, aligning the launch of the platform trial with funders’ timelines, estimating budgets for future arms, and unequal benefits to institutions and individual careers. The need for specific, flexible, ongoing structural funding options to maintain platforms was stressed. Respondents also found it important that funding promotes collaboration and enables platform trials to be multi-institutional, instead of a single institution receiving all the funding.

#### Clinical Trial Registries

Of the 40 respondents, 15 (38%) reported challenges at this stage. Clinical trial registries are not set up to easily capture information for each component of platform trials. Respondents were unable to enter complex platform trial information, specifically the status of each arm, specific dates when arms started and stopped, and the dates and number of patients recruited for each arm. They were also unable to define end points for separate arms and upload data for separate arms because clinical trial registries currently do not provide these options. The lack of flexibility of registries often forced investigators to invent figures and dates to meet registry requirements. Respondents saw the need for registries to be updated to be made more flexible to accommodate platform trials.

#### Patient Information and Consent

Of the 40 respondents, 10 (25%) reported challenge at this stage. Platform trials were reported to require more complex patient information sheets and consent forms. Multiple consents for different domains of the trial, or reconsent for each subprotocol added, was reported to confuse and annoy potential trial participants. The development of guidelines on how to best set up platform trial consent forms, generic templates, and strong patient and public involvement were recommended.

#### Data Analysis

Of the 40 respondents, 9 (23%) reported challenges at this stage. Data analysis for platform trials was reported to be much more demanding than traditional trials given the volume of data and the time required for data harvest, cleaning, and monitoring. Determining how to ensure adequate funding to be allocated to statisticians to support data analysis, and guidance for handling shared controls, were suggested as possible solutions by respondents.

#### Results Dissemination

Of the 40 respondents, 8 (20%) reported challenges at this stage. It was reported that it can be challenging to disseminate the results from one comparison of a platform trial while other arms are ongoing. Journal editors and reviewers were also often not yet sufficiently familiar with platform trials, were concerned about adaptive platform trials as a concept, and had strong preferences for more standard trial designs. It was suggested that there needs to be a clear differentiation between trial arms and research comparisons, and that having specific publication guidelines for platform trial protocols would be helpful.

## Discussion

In this survey study with a mixed methods design including multiple platform trials teams, respondents identified a systemic problem of implementing platform trials because of the fundamental conflict with the current clinical research landscape, which is primarily set up for traditional RCTs. This can lead to substantial challenges conducting platform trials, including unsuitable funding (for the platform trial infrastructure and for joining existing platform trials), high operational complexity, and overburdened staff. As platform trials are now an accepted part of the clinical research, the trial toolbox and process need to be adapted. For instance, one unique finding of this study, which is symptomatic of this conflict and not previously identified, is how clinical trial registers are not set up well to capture information about the multiple comparisons and plans of platform protocols. This is problematic and requires work by each of the national and international registers. This likely applies to other innovative trial designs using master protocols such as basket or umbrella trials or trials with adaptive features such as response-adaptive randomization. In general, all regulatory authorities should be familiarized with the concepts of innovative trial designs and work with researchers to adapt the submission process accordingly.

Another important finding of the study is the lack of consistency on whether the initial investment in platform trials would ultimately save costs and resources when adding a new arm, compared with initiating a new traditional trial. This is somewhat surprising because one of the main arguments to invest in the complex trial infrastructure is the anticipated long-term savings of resources when assessing additional medical interventions within randomized comparisons.^10,11^

A systematic review of existing platform trials suggested that approximately 40% of platform trials actually never added a new treatment comparison, indicating that in some cases planning a traditional parallel-group RCT might have been sufficient.^8^ Hence, careful consideration is required to determine whether such a comparison should better be conducted in a traditional RCT or assessed as part of a multicomparisons platform trial. Platform trials should likely be the design of choice in situations where there is an urgent need for prompt evidence generation about several interventions (eg, in a pandemic situation or for a disease or condition with a high likelihood that there will be more potential treatment options becoming available for evaluation in the future).

### Limitations

This study should be taken in the context of both its strengths and limitations. We received responses from a total of 40 experts, representing 36 different platform trials. Because the survey could be forwarded by invited experts to further experts or team members, we do not know the exact response rate. This approach was chosen to include a wide range of global experts who have direct experience with platform trials, which makes it likely that this study has captured key aspects of a multisided issue. Nevertheless, given that we distributed 138 invitations (127 to principal investigators of platform trials and 11 to researchers with methodological expertise), the response rate was certainly below 30%. This low and rather uncertain response rate is the main limitation of our study, as the sample may not be fully representative of all platform trials. Specifically, because no responses were received from representatives of industry sponsored platform trials (eTable 1 in Supplement 1) challenges and facilitators specific to these trials may have been missed. Given the low response rate, there may be response bias, which could have led to an overrepresentation of stronger or more critical opinions as well as more socially desirable responses.^23,24,25^ Furthermore, several of the respondents might have contributed to other studies in this setting, so these findings should not be seen as entirely independent from these past studies.^12,13,14,15,16^ Even though we invited principal investigators from all globally identified platform trials, some answers might be country-specific. Nevertheless, because many of the key issues are associated with aspects that are common in many countries (eg, incompatible processes, limited resources available, and incompatibility of trial registries), these findings are likely to be of global interest. In addition, respondents held a variety of roles, and some challenges were likely role dependent. A further limitation is that the selected platform trials were identified in 2022; hence, newly initiated platform trials were not considered.^9^

## Conclusions

In this survey study of platform trial experts, it was found that the clinical research landscape is not adapted to facilitate adaptive platform trials, which causes unnecessary challenges. The clinical research community needs to consider how to better integrate platform trials so they can be appropriately and efficiently conducted when needed.

## Notes

Supplement 1. eAppendix. Survey eTable 1. Baseline characteristics of the 127 platform trials contacted and the 32 with survey responses eTable 2. Platform trials vs. traditional randomized trials eTable 3. Number of challenges as mentioned by experts during the survey eTable 4. Number of proposed facilitators as mentioned by experts during the survey

Supplement 2. Data Sharing Statement
