# Supplementary material for: Barriers and Facilitators of Platform Trials
Source: JAMA Netw Open. 2026 Apr 2;9(4):e263758. doi: 10.1001/jamanetworkopen.2026.3758 (PMC13047460; doi:10.1001/jamanetworkopen.2026.3758)
Supplement: Supplement 1. — eAppendix. Survey eTable 1. Baseline characteristics of the 127 platform trials contacted and the 32 with survey responses eTable 2. Platform trials vs. traditional randomized trials eTable 3. Number of challenges as mentioned by experts during the survey eTable 4. Number of proposed facilitators as mentioned by experts during the survey [file jamanetwopen-e263758-s001.pdf]

Supplemental Online Content

McLennan S, Griessbach A, Love S, et al. Barriers and facilitators of platform trials. *JAMA Netw. Open*. 2026;9(4):e263758. doi:10.1001/jamanetworkopen.2026.3758

eAppendix. Survey

eTable 1. Baseline characteristics of the 127 platform trials contacted and the 32 with survey responses

eTable 2. Platform trials vs. traditional randomized trials

eTable 3. Number of challenges as mentioned by experts during the survey

eTable 4. Number of proposed facilitators as mentioned by experts during the survey

This supplemental material has been provided by the authors to give readers additional information about their work.

© 2026 McLennan S et al. *JAMA Network Open*.

eAppendix. Survey

A A A

Identifying and Addressing the Challenges of Platform Trials

Dear Investigator,

Our teams at the University Hospital Basel and MRC CTU at University College London, would like to document your experiences with regard to the challenges of planning and conducting platform trials.

The survey will take approximately 15 minutes. You can save your responses and return at a later date by clicking "*save and return later*" and entering your e-mail address.

We look forward to your feedback and if any questions arise, please do not hesitate to contact us at alexandranatacha.griessbach@usb.ch

Yours sincerely,

Prof. Matthias Briel
Prof. Matt Sydes
Dr. Sharon Love
PhD cand. Alexandra Griessbach

0. General Information

Please enter the *name* or *acronym* of the platform trial(s) you are or have been involved in?

number of PTs

© 2026 McLennan S et al. *JAMA Network Open*.

In what tasks are you involved in? (tick all that apply)

- ☐ Principal/Chief Investigator or Supervisor
- ☐ Trial management
- ☐ Statistics and trial methodology
- ☐ Monitoring
- ☐ Regulatory affairs
- ☐ Data management and programming
- ☐ PPI
- ☐ Site Investigator
- ☐ Other

Do you have central and/or site specific expertise?

○ Central organisational expertise ○ Site specific expertise ○ Both

1. Did you come across any challenges specific to platform trial in these areas?

Please only describe challenges specific to platform trials or tasks which are made more difficult due to the platform trial design.

Regulatory Processes for platform trials ◉ Yes ○ No

(e.g. ethics approval, approval of other authorities, amendments, adding of arms or subprotocols, market approval, communication with regulatory authorities, etc.)

Please describe the challenges in more detail.

What should change? Do you have any ideas how these challenges could be overcome?

Funding Acquisition for platform trials ◉ Yes ○ No

(e.g. funding by different partners and for different arms)

© 2026 McLennan S et al. *JAMA Network Open*.

Please describe the challenges in more detail.

What should change? Do you have any ideas how these challenges could be overcome?

Planning and Setup of platform trials

(e.g. site setup specific to platform trials, stakeholder coordination, contracts, setup of infrastructure, organisation of trial committees, international setup)

◉ Yes ○ No

Please describe the challenges in more detail.

What should change? Do you have any ideas how these challenges could be overcome?

Informed Consent, Patient Information and Patient and Public Involvement (PPI) for platform trials

(e.g. informing patient about platform trial, informed consent update when arms change)

◉ Yes ○ No

Please describe the challenges in more detail.

What should change? Do you have any ideas how these challenges could be overcome?

© 2026 McLennan S et al. *JAMA Network Open*.

Interaction with Clinical Trial Registries

(e.g. clinicaltrials.gov, EUDRACT, WHO clinical trial registry, ACTRN etc.)

(●) Yes ( ) No

Please describe the challenges in more detail.

What should change? Do you have any ideas how this barrier could be overcome?

Conduct of platform trial

(e.g. recruitment to many arms, new sites added to platform trial, stakeholder coordination during conduct, data management, monitoring, adding and dropping of arms or subprotocols, trial committees etc.)

(●) Yes ( ) No

Please describe the challenges in more detail.

What should change? Do you have any ideas how these challenges could be overcome?

Data Analysis for platform trials

(e.g. interim analyses, data cleaning, statistical analysis, adaptive analyses)

(●) Yes ( ) No

Please describe the challenges in more detail.

© 2026 McLennan S et al. *JAMA Network Open*.

**What should change? Do you have any ideas how these challenges could be overcome?**	
**Results Dissemination for platform trials**<br>(e.g. manuscript preparation, publication, review process and conferences for platform trial arm results)	(●) Yes ( ) No
**Please describe the challenges in more detail.**	
**What should change? Do you have any ideas how these challenges could be overcome?**	
**Are there any Other challenges you would like to describe?**	(●) Yes ( ) No
**Please describe the challenges in more detail.**	
**What should change? Do you have any ideas how these challenges could be overcome?**	

2. Advantages of Platform Trials in comparison to traditional randomised controlled trials (RCTs)

In your opinion, name the most important advantage of platform trials? (apart from adding and stopping arms)

© 2026 McLennan S et al. *JAMA Network Open*.

3. Please rate the following platform trial features in comparison to traditional randomised controlled trials (RCTs)

Please choose the value you most agree with.

The planning of a new platform trial takes _____ than the planning of a traditional 2-arm RCT.

○ much less time ○ less time ○ equal amount of time ○ more time ○ much more time
○ unsure

The planning of adding a new comparison to a platform trial takes _____ than planning a standalone RCT.

○ much less time ○ less time ○ equal amount of time ○ more time ○ much more time
○ unsure

Study team expertise and training required for a platform trials is _____ than the expertise needed for traditional RCTs.

○ much lower ○ lower ○ equal ○ higher ○ much higher ○ unsure

The costs and resources needed for one comparison within a platform trial is _____ compared to a traditional RCT.

○ much lower ○ lower ○ equal ○ higher ○ much higher ○ unsure

The time until the first patient is randomized into a new platform trial arm is _____ than the time needed to randomize the first patient into a RCT.

○ much less ○ less ○ equal ○ more ○ much more ○ unsure

4. Teaching and Training for Platform Trials

Do you think there is a need for training in platform trial statistics, operations and ethics? (tick all that apply)

☐ No

© 2026 McLennan S et al. *JAMA Network Open*.

- [ ] Yes, statistical considerations
- [ ] Yes, operational aspects
- [ ] Yes, ethical aspects
- [ ] Don't know

5. Interest in Interview or Focus Group Discussion

In future, would you be willing to participate in an interview or focus group, with the aim to explore the barriers and facilitators of platform trials in more detail?

◯ Yes

◯ No

Any further comments?

Submit

Save & Return Later

© 2026 McLennan S et al. *JAMA Network Open*.

eTable 1: Baseline characteristics of the 127 platform trials contacted and the 32 with survey responses

	127 platform trials[a]	32 platform trials from which at least one team member responded[b]
**Sponsor**		
Non-industry	37 (29.1%)	32 (100.0%)
Industry	90 (70.9%)	0 (0.0%)
**Medical Field**		
COVID trials	45 (35.4%)	16 (50.0%)
Oncology trials	57 (44.9%)	8 (25.0%)
Other	25 (19.7%)	8 (25.0%)
**Planned sample size**		
<100	4 (3.1%)	0 (0%)
100-1000	65 (51.2%)	13 (40.6%)
>1000	41 (32.3%)	16 (50.0%)
not reported	17 (13.4%)	3 (9.4%)
**Funder**		
Public	57 (44.9%)	23 (71.9%)
Industry	36 (28.3%)	0 (0%)
both	34 (26.8%)	9 (28.1%)
**Trial phase**		
Early phase	55 (43.3%)	8 (25.0%)
Late phase	47 (37.0%)	16 (50.0%)
both	25 (19.7%)	8 (25.0%)
**Intervention type**		
Drug	108 (85.0%)	26 (81.3%)
Non-drug	8 (6.3%)	2 (6.3%)
both	11 (8.7%)	4 (12.5%)
**Multicentre or single centre**		
Single centre	7 (5.5%)	1 (3.1%)
Multicentre and national	53 (36.2%)	12 (37.5%)
Multicentre and international	74 (58.3%)	19 (59.4%)

[a]Contacted all currently known platform trials from 2022 (*Griessbach et al., JAMA Netw Open 2024*)
[b]Responders were also involved in 4 platform trials which were not identified in the systematic review; no baseline characteristics available

© 2026 McLennan S et al. *JAMA Network Open*.

eTable 2: Platform trials vs. traditional randomized trials

**Answer options**	**Questions**				
	**Planning a platform trial compared to a traditional 2-arm RCT takes…**	**Time needed until the first patient is recruited into a new platform trial arm compared to recruiting a patient into a new trial takes…**	**The planning of adding a new comparison to a platform trial compared to designing a new standalone trial takes…**	**Study team expertise and training required for platform trials compared to traditional RCTs is…**	**The costs and resources needed for a platform trial comparison compared to a traditional RCT are….**
…much more time/much higher	18 (45%)	3 (8%)	3 (8%)	11 (28%)	3 (8%)
…more time/higher	15 (38%)	12 (30%)	5 (13%)	20 (50%)	6 (15%)
…equal amount of time/equal	1 (2%)	9 (23%)	4 (10%)	5 (13%)	10 (25%)
…less time/lower	1 (2%)	7 (18%)	17 (43%)	0 (0%)	11 (28%)
…much less time/much lower	0 (0%)	2 (5%)	3 (8%)	0 (0%)	1 (2%)
Unsure	2 (5%)	4 (10%)	5 (13%)	2 (5%)	5 (13%)
Missing answer	3 (8%)	3 (8%)	3 (8%)	2 (5%)	4 (10%)

Abbreviations: RCT=randomized controlled trial

© 2026 McLennan S et al. *JAMA Network Open*.

eTable 3: Number of challenges as mentioned by experts during the survey (assessed by study authors based on provided quotes)

Survey question	Codes	N
Regulatory Processes for platform trials	Lack of Expertise	8
Regulatory Processes for platform trials	Problems with adding new arms	4
Regulatory Processes for platform trials	Increased regulatory complexity	3
Regulatory Processes for platform trials	Regulatory process incompatible with platform trial design	3
Regulatory Processes for platform trials	Time-Intensive	3
Regulatory Processes for platform trials	Not all sites participating in all arms	2
Regulatory Processes for platform trials	Online regulatory infrastructure incompatible with platform trial design	2
Regulatory Processes for platform trials	Challenges in International Setting	1
Regulatory Processes for platform trials	Complex legal framework	1
Regulatory Processes for platform trials	Increased complexity in trial conduct	1
Regulatory Processes for platform trials	Master protocol approval not possible without subprotcol	1
Regulatory Processes for platform trials	Online registry infrastructure incompatible with platform trial design	1
Regulatory Processes for platform trials	Process not defined for adding arms	1
Regulatory Processes for platform trials	delayed conduct	1
Funding Acquisition for platform trials	Large & longterm funds needed	6
Funding Acquisition for platform trials	Funding new arms	5
Funding Acquisition for platform trials	Coordination of multiple funders	4
Funding Acquisition for platform trials	Funding infrastructure	4
Funding Acquisition for platform trials	Budgeting of Platform trials	3
Funding Acquisition for platform trials	Public Funding process incompatible with platform trial design	3
Funding Acquisition for platform trials	Budget/funding tool incompatible with platform trial design	2
Funding Acquisition for platform trials	Insufficient non-commercial funding for intervention arms	2
Funding Acquisition for platform trials	Uncertainty about cost savings	2
Funding Acquisition for platform trials	Complex legal framework (contracts)	1
Funding Acquisition for platform trials	Funding Platform trial planning	1
Funding Acquisition for platform trials	Shared credit for several platform PIs not possible	1
Planning and Setup of platform trials	Complex legal framework (contracts)	8
Planning and Setup of platform trials	High operational complexity	5
Planning and Setup of platform trials	Lack of Expertise	5
Planning and Setup of platform trials	Challenges in International Setting	4
Planning and Setup of platform trials	Challenging site set-up	4
Planning and Setup of platform trials	Complex IMP Management and Storage	4
Planning and Setup of platform trials	delayed conduct	4
Planning and Setup of platform trials	Conflict of intrest with multiple stakeholder	3
Planning and Setup of platform trials	Extensive Management of Committees	3
Planning and Setup of platform trials	Complex database set-up	3
Planning and Setup of platform trials	Increased regulatory complexity	3
Planning and Setup of platform trials	Large & longterm funds needed	2
Planning and Setup of platform trials	Not all sites participating in all arms	2

© 2026 McLennan S et al. *JAMA Network Open*.

Survey question	Codes	N
Planning and Setup of platform trials	Complex statistics	2
Planning and Setup of platform trials	Complexities with large study teams	2
Planning and Setup of platform trials	Challenges due to uncertaintes about platform progression	1
Planning and Setup of platform trials	Complex legal framework (contracts) /Funder	1
Planning and Setup of platform trials	High administrative burden	1
Planning and Setup of platform trials	Insurance process incompatible with platform trial design	1
Planning and Setup of platform trials	Online registry infrastructure incompatible with platform trial design	1
Informed Consent, Patient Information and Patient and Public Involvement (PPI) for platform trials	Designing Patient-Friendly IC challenging	4
Informed Consent, Patient Information and Patient and Public Involvement (PPI) for platform trials	High information burden on patients	4
Informed Consent, Patient Information and Patient and Public Involvement (PPI) for platform trials	Time consuming preparation of IC (stakeholder involvement, video etc) needed	3
Informed Consent, Patient Information and Patient and Public Involvement (PPI) for platform trials	Modular design (multiple consents for arms) burdensome	3
Informed Consent, Patient Information and Patient and Public Involvement (PPI) for platform trials	Modular design (multiple consents for arms) error prone	1
Informed Consent, Patient Information and Patient and Public Involvement (PPI) for platform trials	Regular amendments required	1
Informed Consent, Patient Information and Patient and Public Involvement (PPI) for platform trials	Lack of Expertise	1
Interaction with Clinical Trial Registries	Online infrastructure incompatible with platform trial design	11
Interaction with Clinical Trial Registries	No sample size (planned and actual) for seperate arms	4
Interaction with Clinical Trial Registries	No start and end date for seperate arms	3
Interaction with Clinical Trial Registries	multiple domains/arms as a single entry or multiple entries	3
Interaction with Clinical Trial Registries	Uploading/publishing results for completed arms while platform ongoing	2
Interaction with Clinical Trial Registries	Different effect measures for different arms	1
Interaction with Clinical Trial Registries	Lack of Expertise	1
Interaction with Clinical Trial Registries	No status for seperate arms	1
Interaction with Clinical Trial Registries	Registry not user friendly	1
Interaction with Clinical Trial Registries	Uploading results for multiple arms	1
Interaction with Clinical Trial Registries	Uploading/publishing results for multiple arms	1
Conduct of platform trial	Overburdened staff/ Understaffing	7
Conduct of platform trial	Open-end study burdensome	6
Conduct of platform trial	Adding new arms	7
Conduct of platform trial	High operational complexity	6
Conduct of platform trial	Lack of Expertise	6
Conduct of platform trial	Blinding challenge	3
Conduct of platform trial	Complex data management	3
Conduct of platform trial	Increased regulatory complexity	2
Conduct of platform trial	Multiple stakeholders have different expectations on the conduct	2
Conduct of platform trial	Complex IMP Management and Storage	2
Conduct of platform trial	Shared controls	2
Conduct of platform trial	Challenges in International Setting	1
Conduct of platform trial	Challenging site set-up	1
Conduct of platform trial	Complex legal framework (contracts)	1

© 2026 McLennan S et al. *JAMA Network Open*.

Survey question	Codes	N
Conduct of platform trial	Complex randomisation	1
Conduct of platform trial	Complex statistics	1
Conduct of platform trial	Extensive Management of Committees	1
Conduct of platform trial	Funding new arms	1
Conduct of platform trial	High Data Volume	1
Conduct of platform trial	High Monitoring Burden	1
Conduct of platform trial	High staff turnover	1
Conduct of platform trial	IT challanges?	1
Conduct of platform trial	Recruitment problems	1
Conduct of platform trial	Regulatory process incompatible with platform trial design	1
Conduct of platform trial	Risk of neglegting reviews/checks when adding a new arm	1
Conduct of platform trial	Uncertainty about cost savings	1
Conduct of platform trial	increased risk for disapointment due to multiple arms	1
Data Analysis for platform trials	Complex statistics	5
Data Analysis for platform trials	Complex data management	3
Data Analysis for platform trials	High Data Volume	2
Data Analysis for platform trials	Lack of Expertise	2
Data Analysis for platform trials	Adequate reporting	1
Data Analysis for platform trials	Blinding challenge	1
Data Analysis for platform trials	High Monitoring Burden	1
Data Analysis for platform trials	High complexity when recruiting patients who are not eligible for all arms	1
Data Analysis for platform trials	Lack of expertise	1
Data Analysis for platform trials	Shared control	1
Data Analysis for platform trials	Shared controls	1
Results Dissemination for platform trials	Multiple manuscripts from same platform trial in circulation	2
Results Dissemination for platform trials	Overburdened staff/ Understaffing	2
Results Dissemination for platform trials	Uploading/publishing results for completed arms while platform ongoing	2
Results Dissemination for platform trials	Lack of Expertise	1
Results Dissemination for platform trials	Shared controls	1
Results Dissemination for platform trials	lack of guidance on Protocol publication	1
Are there any other challenges you would like to describe?	Adding new arms	1
Are there any other challenges you would like to describe?	Collaboration with industry	1
Are there any other challenges you would like to describe?	Important that platform does not halt	1

© 2026 McLennan S et al. *JAMA Network Open*.

eTable 4: Number of proposed facilitators as mentioned by experts during the survey (assessed by study authors based on provided quotes)

**Survey question**	**Codes**	**N**
Regulatory Processes for platform trials	Modification of regulations	5
Regulatory Processes for platform trials	NONE*	5
Regulatory Processes for platform trials	More experience over time	3
Regulatory Processes for platform trials	Modification of CTIs	2
Regulatory Processes for platform trials	New guidance (regulatory affairs)	3
Regulatory Processes for platform trials	Centralized ethical approval	1
Regulatory Processes for platform trials	Education and Training	1
Regulatory Processes for platform trials	Ethic committees specialized for platform trials	1
Regulatory Processes for platform trials	Fast track for adding new arms	1
Regulatory Processes for platform trials	Internaltional Collaboration amongst EC	1
Regulatory Processes for platform trials	New Guidance (trial set-up & management)	1
Regulatory Processes for platform trials	Specialized teams at investigator level	1
Funding Acquisition for platform trials	NONE*	5
Funding Acquisition for platform trials	New funding process needed	5
Funding Acquisition for platform trials	Infrastructure /set-up grants	4
Funding Acquisition for platform trials	Standard cost templates	2
Funding Acquisition for platform trials	funding for new arms	2
Funding Acquisition for platform trials	Adapt budget/funding tools	1
Funding Acquisition for platform trials	Allowing to have different PIs per platform (for each comparison), who can receive funding	1
Funding Acquisition for platform trials	Contracts flexible to include future stakeholders	1
Funding Acquisition for platform trials	Education and Training (budgeting/ costing)	1
Funding Acquisition for platform trials	Exchange of experienced stakeholders	1
Funding Acquisition for platform trials	Flexible funding needed for fast adaptations	1
Funding Acquisition for platform trials	More non-commercial funding	1
Funding Acquisition for platform trials	Seed funding grants for platform trials	1
Funding Acquisition for platform trials	funding for international expansion	1
Planning and Setup of platform trials	NONE*	12
Planning and Setup of platform trials	Early clarification of roles and expectations	3
Planning and Setup of platform trials	Early communication with stakeholders/trial team	2
Planning and Setup of platform trials	Faster legal contracts	2
Planning and Setup of platform trials	Specific templates for contracts	2
Planning and Setup of platform trials	Centralized ethical approval	1
Planning and Setup of platform trials	Education and Training	1
Planning and Setup of platform trials	Education and Training (logistics-IMPs)	1
Planning and Setup of platform trials	Education and Training (EC/Authorithies)	1
Planning and Setup of platform trials	Education and Training (Site Investigator)	1
Planning and Setup of platform trials	Modification of regulations	1
Planning and Setup of platform trials	More experience over time	1
Planning and Setup of platform trials	More flexible governance	1
Planning and Setup of platform trials	New Guidance (trial committees)	1
Planning and Setup of platform trials	New Guidance (governance, regulatory affairs)	1
Planning and Setup of platform trials	New Guidance (simulation and sample size calculations)	1

© 2026 McLennan S et al. *JAMA Network Open*.

Survey question	Codes	N
Planning and Setup of platform trials	New Guidance (trial set-up & management)	1
Planning and Setup of platform trials	New guidance (regulatory affairs in different countries)	1
Planning and Setup of platform trials	Specialized teams covering needed competences	1
Planning and Setup of platform trials	Standadized templates	1
Planning and Setup of platform trials	Standadized templates (for Industry-non-industry collaboration)	1
Planning and Setup of platform trials	Sufficient time for planning	1
Planning and Setup of platform trials	Transparent rules for data handling and analysis	1
Planning and Setup of platform trials	Use established partnerships	1
Planning and Setup of platform trials	infrastruture/set-up grants	1
Informed Consent, Patient Information and Patient and Public Involvement (PPI) for platform trials	Implementation of PPI in platform tials	3
Informed Consent, Patient Information and Patient and Public Involvement (PPI) for platform trials	Specific template for modular patient information & consent forms.	3
Informed Consent, Patient Information and Patient and Public Involvement (PPI) for platform trials	NONE*	2
Informed Consent, Patient Information and Patient and Public Involvement (PPI) for platform trials	Better allignement of processes in clinical routine and clinical trials	1
Informed Consent, Patient Information and Patient and Public Involvement (PPI) for platform trials	Evaluating impact of PPI involvment	1
Informed Consent, Patient Information and Patient and Public Involvement (PPI) for platform trials	New Guidance (IC for platform trials)	1
Informed Consent, Patient Information and Patient and Public Involvement (PPI) for platform trials	New Guidance (PPI involvement)	1
Interaction with Clinical Trial Registries	Restructuring of registry to accomodate platform trials (be more flexible)	7
Informed Consent, Patient Information and Patient and Public Involvement (PPI) for platform trials	PPI in trial board needed	1
Interaction with Clinical Trial Registries	NONE*	4
Interaction with Clinical Trial Registries	Ability to enter a sample size or status per treatment arm	1
Interaction with Clinical Trial Registries	Ability to enter multiple domains	1
Interaction with Clinical Trial Registries	Ability to enter results per treatment arm	1
Interaction with Clinical Trial Registries	Allow linking of subprotocols	1
Interaction with Clinical Trial Registries	Trial registries should be intuitive/user-friendly	1
Conduct of platform trial	NONE*	8
Conduct of platform trial	Training and education	5
Conduct of platform trial	Specialized teams covering needed competences	3
Conduct of platform trial	Sufficient resources for project management	3
Conduct of platform trial	Appropriate budgeting	2
Conduct of platform trial	Specific plans for pat recruitment and working conditions on-site (e.g. recruitment breaks)	2
Conduct of platform trial	Sufficient resources for adding arms	2
Conduct of platform trial	Allowing to consenting only for a selection of treatment arms	1
Conduct of platform trial	Blinded and unblinded statistical teams	1

Survey question	Codes	N
Conduct of platform trial	Centralized and remote Monitoring	1
Conduct of platform trial	Increased support of study team on-site	1
Conduct of platform trial	More experience over time	1
Conduct of platform trial	More non-commercial grants for platform trials	1
Conduct of platform trial	New Guidance (trial Committee members)	1
Conduct of platform trial	New guidance (regulatory affairs)	1
Conduct of platform trial	Specific template for modular patient information & consent forms.	1
Conduct of platform trial	Sufficient time for planning	1
Conduct of platform trial	Training and Education	1
Conduct of platform trial	Validation checks for CRF when adding new arms	1
Conduct of platform trial	Infrastructure /set-up grants	1
Data Analysis for platform trials	NONE*	5
Data Analysis for platform trials	Minimize data collection	1
Data Analysis for platform trials	New Guidance (shared controls)	1
Data Analysis for platform trials	Sufficient resources for data analysis	1
Results Dissemination for platform trials	NONE*	5
Results Dissemination for platform trials	Education and Training	1
Results Dissemination for platform trials	New Guidance (publication of master and sub protocols)	1
Are there any other challenges you would like to describe?	Advertising to industry to test new interventions	1
Are there any other challenges you would like to describe?	Potential new arms already prepared to continue platform trial	1

*Meaning the respondent stated a specific challenge but did not suggest a facilitator (i.e. did not fill provide a value for the question "*What should change? Do you have any ideas how these challenges could be overcome?")*

© 2026 McLennan S et al. *JAMA Network Open*.
