# Supplementary material for: Barriers and Facilitators of Platform Trials
Source: JAMA Netw Open. 2026 Apr 2;9(4):e263758. doi: 10.1001/jamanetworkopen.2026.3758 (PMC13047460; doi:10.1001/jamanetworkopen.2026.3758)
Supplement: Supplement 2. — Data Sharing Statement [file jamanetwopen-e263758-s002.pdf]

Data Sharing Statement

McLennan. Barriers and Facilitators of Platform Trials. *JAMA Netw Open*. Published April 02, 2026. doi:10.1001/jamanetworkopen.2026.3758

Data
Data available: No

Additional Information
Explanation for why data not available: Survey respondents were informed that their responses would be treated confidentially and that they would not be identifiable. Responses (e.g., free-text answers) contain information that could potentially reveal the identity of the respondents.
